# Psychometric properties of the Chinese mainland version of the Palliative Care Spiritual Care Competency Scale (PCSCCS-M) in nursing: a cross-sectional study

**DOI:** 10.1186/s12904-019-0409-6

**Published:** 2019-03-08

**Authors:** Yanli Hu, Fan Li, Jeng-Fong Chiou

**Affiliations:** 1 School of Nursing, Jilin University, Changchun, China; 2 Department of Pathogenobiology, The Key Laboratory of Zoonosis Research, Chinese Ministry of Education, College of Basic Medicine, Jilin University, No. 126 Xinmin Street, Changchun, 130021 China; 3 The Key Laboratory for Bionics Engineering, Ministry of Education, Jilin University, Changchun, Jilin China; 4 State Key Laboratory of Pathogenesis, Prevention and Treatment of High Incidence Diseases in Central Asia, Urumqi, Xinjiang China; 5 Department of Radiology, School of Medicine, College of Medicine, Taipei Medical University, Taipei, Taiwan; 6 Department of Radiation Oncology, Taipei Medical University Hospital, Taipei, Taiwan

**Keywords:** Spiritual care, Competency, Nurses, Validity and reliability

## Abstract

**Background:**

Spiritual care competencies are among the primary professional skills that enable best practices in nursing. Assessing these competencies and identifying those that are insufficient are important tasks. The traditional Chinese version of the Palliative Care Spiritual Care Competency Scale (PCSCCS) used in Taiwan is a well-validated tool to measure palliative caregivers’ competencies in providing spiritual care. However, whether this scale is valid and reliable for use with nurses in other health-care contexts is unknown. The purpose of this study is to determine this version’s validity and reliability for use with nurses in mainland China.

**Methods:**

The PCSCCS was first converted into a simplified Chinese version (PCSCCS-M) from the traditional Chinese version used in Taiwan such that mainland nurses could read and understand it easily. Then, the validity and reliability of the PCSCCS-M was evaluated with 400 Chinese nurses recruited using convenience sampling from three university-affiliated comprehensive hospitals, two cancer hospitals, one psychiatric hospital, two traditional Chinese medicine hospitals, one marital and child service care center, and one community health service center. Concurrent validity was assessed using Pearson’s correlation coefficients of the PCSCCS-M and the Chinese version of the Spiritual Care-Giving Scale (C-SCGS). Exploratory factor analysis (EFA) was performed to determine the construct validity. Confirmatory factor analysis (CFA) was conducted using another sample of 351 nurses to verify the quality of the factor structures of the PCSCCS-M. An internal consistency test based on Cronbach’s alpha coefficient and a stability test based on the Guttman split-half coefficient were also conducted.

**Results:**

Useful data were obtained from 356 participants (response rate: 89%). EFA confirmed a three-dimensional structure of the scale after one item was deleted, and the three factors explained 63.839% of the total variance. Cronbach’s alpha coefficients of the three subscales were 0.811, 0.889 and 0.896, and the Guttman split-half coefficient for the PCSCCS-M was 0.862. Modified CFA indicated a well-fitting model. The correlation between the PCSCCS-M and C-SCGS was 0.340 (*p* < 0.01).

**Conclusions:**

The PCSCCS-M is a brief, easy-to-understand, and psychometrically sound measurement tool to evaluate spiritual care competencies in nurses from mainland China.

**Electronic supplementary material:**

The online version of this article (10.1186/s12904-019-0409-6) contains supplementary material, which is available to authorized users.

## Introduction

Spiritual care is a core component of health care because fulfillment of patients’ spiritual needs is significantly associated with their physical, psychological, social, and spiritual well-being [1–4] and their satisfaction with the quality of care [4, 5]. Spiritual care decreases the severity of disease symptoms, reduces hopelessness, and facilitates emotional well-being and inner peace [6]. Additionally, spiritual care helps patients find meaning and purpose in life and in adversity [7–10] and encourages them to consider hospice utilization [3] and to avoid futile, aggressive interventions [11]. In contrast, if health care workers do not provide spiritual care to patients that is consistent with their needs, patients are more likely to be at risk of depression, experience less meaning and peace [5], and have poorer physical, psychological, and spiritual well-being [2, 12]. Therefore, to ensure optimal care and patient outcomes, health care professionals and researchers highlight the importance of adequate spiritual care provision in addressing patients’ spiritual distress and needs. Spiritual care is a key element of health care guidelines (such as national palliative care guidelines) [11, 13].

The effectiveness of spiritual care depends largely on adequate preparedness [7]. Nurses are thought to be competent in caring for the spiritual needs of patients [14]. Nursing competencies in this area refer to the ability to provide spiritual care to patients with optimal outcomes by encouraging the development of loving relationships and competently recognizing and responding to patients’ spiritual distress and needs. These needs include identifying meaning, fostering self-worth, faith support, interactions with sensitive listeners and self-expression [15, 16]. Being competent in the delivery of spiritual care determines the effectiveness of care and is regarded as one of nurses’ primary professional skills [17].

One crucial issue to address is which competencies nurses should acquire to provide optimal spiritual care, the current level of these capacities, and how they can be evaluated. Therefore, testing nurses’ existing competency levels to determine which aspects need to be improved is an important task. Addressing this issue effectively will help nurses and managers determine appropriate further actions and enable them to explore available resources to improve care and patient satisfaction [15–17]. In certain countries, several instruments have been developed to evaluate these competencies, such as the Spiritual Care Competency Scale (SCCS) [18], the Nurses’ Professional Competence in Spiritual Care [19, 20], the Student Survey of Spiritual Care (SSSC) [21], and the Palliative Care Spiritual Care Competency Scale (PCSCCS) [22]. However, such instruments are lacking and cultivation of nurses’ spiritual care ability is currently not valued in mainland China possibly because of the lack of necessary nursing education courses and appropriate teaching resources, such as qualified teachers and teaching content [23]. In China, research on spiritual care education in health care is hindered by the limited number of valid studies and the lack of established measurement instruments. Therefore, the development or introduction of a valid and reliable measurement instrument is urgently required for health care providers, patients, and the education sector.

The PCSCCS, a self-reported scale created by Chen and colleagues [22], potentially measures the competencies of palliative care students and professionals in providing spiritual care to patients. Although both the PCSCCS [22] and the SCCS [18], which was developed by Leeuwen. R., examine spiritual care competency, differences exist between them, including the measurement objects on which these two instruments were developed. The PCSCCS was first designed based on item responses collected from participants in a palliative care spiritual care training program conducted by the Schweitzer Christian and Missionary Alliance [22], while the SCCS (27-item) was developed based on nursing students to assess their spiritual care ability [18]. Therefore, the potentially applicable objects may also differ. In addition, the PCSCCS contains fewer items (18-item) and is more streamlined, thus facilitating measurement.

However, a critical question is whether this scale can be used with health care providers from other cultural contexts, such as nurses in mainland China, most of whom are nonreligious votaries and lack a palliative care background. The psychometric properties of the mainland Chinese version of the PCSCCS have never been assessed. Studies have only reported results in Taiwan using the traditional Chinese version [22], which is distinct from the simplified Chinese version used in mainland China. The term “simplified Chinese” [24] is comparable to “traditional Chinese” and originated in the twentieth century on the basis of the official Chinese language. Character simplification was introduced to reduce interethnic and regional differences. Currently, simplified Chinese is used in mainland China and Singapore, and traditional Chinese is used in Taiwan and Hong Kong. After decades of development, the writing and meaning of the two languages have engendered certain differences [25]. To promote regional health care accessibility and facilitate the spiritual care practice of mainland nurses, the current study attempted to translate the Traditional Chinese version of the PCSCCS [22] used in Taiwan into simplified Chinese. Therefore, the current study had two main aims: 1) to convert the traditional Chinese version of the PCSCCS used in Taiwan into simplified Chinese and apply cultural adjustments and 2) to evaluate the validity and reliability of the simplified Chinese version (PCSCCS-M) for use with nurses from mainland China. We hope that our study serves as a reference for the measurement, assessment and development of Chinese nurses’ spiritual care competencies.

## Methods

### Ethical approval

The present study was reviewed and approved by the Institute Review Board of the College of Nursing, Jilin University (access number 2018031103). All participants were informed of the purpose of the study, which followed the principles of voluntary participation and anonymity, and the participants had the right to choose not to participate and to withdraw from the study without any consequence. Data were collected between March and April 2018.

### Participants

Chinese-speaking nurses were invited to participate in this cross-sectional study. A convenience sample of 400 nurses was recruited from ten health care contexts (three university-affiliated comprehensive hospitals, two cancer hospitals, one psychiatric hospital, two traditional Chinese medicine hospitals, one marital and child service care center, and one community health service center). No exclusion criteria were applied.

### The main measurement instrument

#### The Palliative Care spiritual Care scale

The 18-item traditional Chinese version of the PCSCCS created by Chen and colleagues for use in Taiwan is a spiritual care competency measurement instrument designed for palliative care providers. The original PCSCCS developed by Chen et al. [22] includes three different components: knowledge and skills in spiritual care (Cronbach’s alpha 0.81), self-awareness and attitude toward spiritual care (Cronbach’s alpha 0.88), and provision of spiritual care that meets spiritual needs (Cronbach’s alpha 0.85). The PCSCCS has sound psychometric properties for use in Taiwan.

### Additional measurement instruments

Two additional instruments included in the questionnaire were a demographic form and the Chinese version of the Spiritual Care-Giving Scale (C-SCGS). The demographic form consisted of five questions regarding participants’ age, sex, education, working years, and working department. The original 35-item SCGS was developed by Tiew and Creedy [26] to measure nursing students’ and nurses’ perceptions of spirituality and spiritual care. We translated and evaluated the 34-item C-SCGS [27] with Tiew’s permission. The C-SCGS has four core factors: attributes of spiritual care (Cronbach’s alpha 0.941), definitions of spirituality and spiritual care (Cronbach’s alpha 0.852), spiritual perspectives (Cronbach’s alpha 0.836), and spirituality and spiritual care values (Cronbach’s alpha 0.866). The C-SCGS was used to test the validity of the PCSCCS-M.

### Translation procedure

The first step was translation of the traditional Chinese version of the PCSCCS used in Taiwan into the simplified Chinese language used in mainland China. Permission was obtained from the author of the original PCSCCS. Phase I involved two steps: 1) Font conversion: two independent experts, one from Jilin University and one from Naval Medical University, carried out the task of simplifying the traditional Chinese characters of the PCSCCS used in Taiwan into simplified Chinese characters used in mainland China. The two resultant converted versions were reconciled by a person from mainland China who was familiar with traditional Chinese characters. 2) Evaluation of content validity: an expert panel was asked to ensure whether each item fit a four-point Likert scale to determine the content validity of each item and to confirm whether the items were designed properly to create the constructs. The expert panel included one specialist in oncology, one nurse in an intensive care unit, two nursing professors, and one advanced-practice nurse specializing in palliative care. Ambiguous or complex terms were either removed or rephrased until no changes to the Chinese translation were deemed necessary. A content validity of an item (CVI) score above 0.8 was considered valid [28]. The final CVI analysis revealed that 16 of 18 items achieved a CVI above 0.8. Some experts thought that the content of item 13 was not consistent with the culture of mainland China. Regarding item 14, they also thought that nurses encountered difficulty in spending sufficient time with patients and guiding them to open up and state the problem truthfully under the current allocation conditions of nursing human resources in China. However, considering that the original author believed that these two issues were important for spiritual care, we kept these two items for further analysis.

Phase II consisted of two steps: 1) The revised version of the PCSCCS was pilot tested in a convenience sample of 10 nurses in three Jinlin University-affiliated teaching hospitals to evaluate whether the PCSCCS-M was easy to understand and answer. 2) The psychometric properties of the PSCCS-M were evaluated based on item analysis, construct validity, concurrent validity, internal consistency reliability, and split-half reliability. The construct validity of the PSCCS-M was determined through a principal component analysis with varimax rotation. In the current study, Pearson’s correlation coefficients for the PSCCS-M and the C-SCGS were calculated to assess the concurrent validity of the PSCCS-M. To verify the quality of the component structure, we conducted a confirmatory factor analysis (CFA) based on other data obtained from 351 nurses. This sample size was adequate for exploratory factor analysis (EFA) according to the guideline of the Monte Carlo study decision on sample size [29] or Mokkink, L. B. et al. [30]. We also tested the internal consistency and stability of the scale based on Cronbach’s alpha coefficient and the Guttman split-half coefficient, respectively.

### Data collection

A professional platform called SO JUMP was used for data collection [31]. First, the content of the questionnaire was entered into a computer. Then, the questionnaire was sent to individual nurses through WeChat (a total of 17 nurses) and to 4 WeChat chat groups with fixed numbers of nurses (group 1, 52; group 2, 65; group 3, 60; and group 4, 206) via WhatsApp. Before answering the questionnaires, all participants were asked to sign a written consent form.

### Statistical analysis

SPSS 17.0 IBM was used to perform the data analysis. The internal consistency and homogeneity of the PCSCCS-M were assessed using Cronbach’s alpha. The concurrent validity between the PCSCCS-M and the C-SCGS was assessed by Pearson’s correlation coefficient. Item analysis was performed using the following analyses: (a) extreme group comparison (item discrimination, an item should be able to discriminate between the upper 27% and lower 27% scoring groups) [32]; (b) corrected item-total correlations; (c) factor loadings; (d) Cronbach’s alpha if an item was deleted; (e) and communities. Items with a criterial value (CR) < 3.0, a corrected item-total correlation < 0.30, factor loading < 0.45, and a community < 0.20 and whose deletion caused an increase of 0.5 or more in the alpha coefficient for the overall scale were excluded.

The construct validity of the PCSCCS-M was analyzed by EFA. Prior to the EFA, the Kaiser-Meyer-Olkin (KMO) test and Bartlett’s spherical test were performed. The criterion for factor extraction was an eigenvalue > 1.0 and a factor loading > 0.40.

CFA was conducted using AMOS, version 20.0, to further evaluate the validity of the PCSCCS-M.

## Results

### Sample characteristics

A total of 356 (of a possible 400) nurses completed the survey, resulting in a response rate of 89%. All returned questionnaires were suitable for this study. Most nurses were female (*n* = 336, 94.4%), married (68.8%) and undergraduates (73.3%). Their average length of employment was 10.4 years (SD, 8.8). The basic characteristics of the participants are summarized in Additional file 1: Table S1.

### Psychometric analyses

#### Item analysis

The results of the psychometric analysis showed that items 13 (with 2 substandard indicators) and 14 (with 5 substandard indicators) did not meet the item retention criteria (see Additional file 1: Table S2). Based on these results and the results of subsequent interviews with five nurses, item 14 was ultimately deleted.

#### The 17-item PCSCCS-M

The internal consistency analysis of the 17-item PCSCCS-M showed that the average of each item score ranged from 3.57 to 4.11. Except for item 13 (0.344), the adjusted item-total correlations ranged from 0.580 to 0.780. All item-total correlations were 100% positive, with values from 0.437 to 0.813, indicating moderate to strong correlations. The internal consistency of the 17-item PCSCCS-M was good, with a Cronbach’s alpha of 0.931. Therefore, based on the item analysis results, theoretical considerations, and interviews, we decided not to delete any items (see Additional file 1: Table S3).

#### Face validity, construct validity, and concurrent validity

To assess the face validity, the PCSCCS-M was given to 10 nurses from three different types of hospitals to understand how they perceived and interpreted the items. The participants reported that the wording of the PCSCCS-M was clear and that they had little difficulty understanding it. The construct validity of the PCSCCS-M revealed three distinct factors (confirmed by a scree plot; see the Additional file 2: Figure S1), with eigenvalues greater than 1.0 (8.446, 1.346 and 1.060) and factor loadings ranging from 0.496 to 0.794, all greater than 0.450, on all items. Factor 1 included four items related to “knowledge and skills of spiritual care” (items 1, 2, 3, and 4), Factor 2 included six items related to “self-awareness and attitude toward spiritual care” (items 5, 6, 7, 8, 9, and 10), and Factor 3 included seven items related to “spiritual care that meets patients’ spiritual needs” (items 11, 12, 13, 15, 16, 17, and 18). A structure matrix showed that the factor loadings of items 1, 5, and 6 in both Factor 1 and Factor 2 were greater than 0.40, and the factor loadings of items 11 and 12 were greater than 0.40 in both Factor 2 and Factor 3. However, item 1 was included in Factor 1 because the factor loading of item 1 was more important for Factor 1 and its content addressed knowledge of spiritual care. Additionally, the factor loading values of items 5 and 6 in Factor 2 were greater than the factor loading values in Factor 1, and their contents were highly correlated with self-perception. Thus, items 5 and 6 were attributed to Factor 2. The factor loading value of item 11 was greater in Factor 3 (0.601) than that in Factor 2 (0.556), and item 11 was therefore attributed to Factor 3. Although item 12 had a higher factor loading value in Factor 2 (0.546) than that in Factor 3 (0.532), its content pertained to nurses’ provision of spiritual care to meet patients’ spiritual needs; therefore, item 11 was still included in Factor 3 (the items included in each of the four factors are shown in Additional file 1: Table S4). The model could explain 63.839% of the total variance (Additional file 1: Table S3). The percentages of the variance explained for the PCSCCS-M subscales are listed in Additional file 1: Table S4. Regarding the concurrent validity of the PCSCCS-M, the correlation between the PCSCCS-M and the C-SCGS was 0.340 (*p* < 0.01) (Additional file 1: Table S5).

#### Internal consistency reliability, split-half reliability, and model fit

The KMO measure of the PCSCCS-M was 0.936, which was better than the minimal admissible level of 0.50. Bartlett’s test of sphericity was also admissible (*p* < 0.001). The EFA demonstrated that Cronbach’s alpha coefficient for the PCSCCS-M was 0.931. For its three subscales, Cronbach’s alpha coefficients were 0.811, 0.889 and 0.868, respectively (Additional file 1: Table S5). Additional file 1: Table S2 shows the results of the item analysis. The Guttman split-half coefficient of the PCSCCS-M was 0.862. The results of the CFA showed that the goodness-of-fit indices of the adjusted 3-factor model were good **(**Likelihood-ratio χ2/degree of freedom, 2.215; Goodness of fit index (GFI), 0.937; Root mean square residual (RMR), 0.015; Standardized root mean square error of approximation (RMSEA), 0.059; Normed fit index (NFI), 0.938; Non-normed fit index (NNFI, TLI), 0.954; Incremental fit index (IFI), 0.965; and Comparative fit index (CFI), 0.965). Additional file 1: Table S6 shows the various goodness-of-fit indices for the model of the PCSCCS-M. The results of the CFA showed that the goodness-of-fit indices of the adjusted 3-factor model with 16 items due to the deletion of item 13 were similar to those of the adjusted 3-factor model with 17 items (see Additional file 1: Table S6).

## Discussion

The main aims of this study were to translate the English version of the well-validated PCSCCS into Chinese and to examine the psychometric properties of the PCSCCS-M. The participants of this study were recruited from ten different types and levels of hospitals and various departments. Therefore, the results should represent nurses of diverse backgrounds. Overall, the PCSCCS-M showed good face validity, construct validity, and internal consistency in our study. The results showed that three factors corresponded with the findings of Chen et al. Cronbach’s alpha for all three factors was higher than 0.80, providing empirical evidence that the psychometric properties were within an acceptable and ideal range. Furthermore, another set of data from 351 participants was used to conduct a CFA. The results showed that the response data fit reasonably well with the hypothetical structure of the PCSCCS-M, which provided positive evidence for its construct validity. We propose that the PCSCCS-M is an appropriate tool for assessing the competency of spiritual care providers in mainland China.

Compared with the original English version of the PCSCCS, the PCSCCS-M performed well, with Cronbach’s alpha coefficients of 0.811, 0.889 and 0.868 for knowledge and skills of spiritual care (Factor 1), self-awareness and attitude toward spiritual care (Factor 2), and spiritual care that meets spiritual needs (Factor 3), respectively. More than 63% of the total variance could be accounted for by the three-factor model, and the percentage was higher than the percentage of 58.34% observed for the Taiwanese version. The split-half internal consistency test of the scale, with a correlation of 0.862 between the two halves, also demonstrated the sound reliability of the PCSCCS-M. In addition, the PCSCCS-M showed significant concurrent validity with the C-SCGS, indicating that these measures have unique constructs. A minor difference was found between the PCSCCS-M items and the PCSCCS items in a study conducted by Chen et al. [18]: the former consisted of 17 items, and the latter consisted of 18 items. The outcomes of the psychometric analysis revealed that item 13, “It is difficult for me to help patients forgive and feel forgiven”, and item 14, “It is hard for me to get patients to open up and truly state their problems”, were not satisfactory and did not meet the criteria of item retention. However, we considered that obtaining more insight through nurses’ self-assessment of these aspects may help explain this unsatisfactory statistical result and thus promote the validity and reliability of this scale. Therefore, after completing the item analysis, additional interviews were performed by two investigators with five individual nurses recruited using a random method to clarify the content of these two items. The results of these interviews indicated that nurses were generally confused about the precise meaning of item 13. Even if the researchers had explained the item properly, four of five nurses said that they were unfamiliar with the subject and that communicating it to patients was difficult. Three of them said that forgiveness and being forgiven are a patient’s personal business and that nurses’ involvement in such business is inappropriate. One nurse said that most nurses cannot forgive or feel forgiven, let alone help and coach patients to do so. Additionally, nurses demonstrated that they had difficulty scoring themselves on this item. Thus, the content of this item did not appear to measure the current level of spiritual care. However, forgiveness therapy plays a vital role in patients’ physical and mental health recovery [33–35], and the results of the CFA of the adjusted 3-factor model with 16 items due to the deletion of item 13 showed no significant improvement in the fitting indicators (Additional file 1: Table S6); therefore, we decided to retain this item in the scale with the recommendation of providing relevant examples to illustrate concepts of self-forgiveness, forgiving others, and feeling forgiveness from God. For item 14, three nurses indicated that the content was easy to understand but difficult to accomplish both subjectively and objectively mainly because nurses have limited time to communicate with patients due to a shortage of nurses. In addition, some nurses implied that the current situation of the medical treatment environment has some negative influences on patient-nurse relationships and that misunderstandings occur between health workers and patients. Therefore, to protect themselves, nurses are not willing to discuss problems other than those related to health care with patients. Because this status quo could not be changed in a short time, this item was temporarily deleted from the scale in the present study. However, considering that the contents of these items are meaningful to patients, we recommend that future researchers apply appropriate adjustments and retest these items. Moreover, with the development of spiritual care education, caregivers’ understanding of spiritual care is also deepening; thus, future measurements should include items 13 and 14. We recommend that future studies retain these two items as appropriate on the basis of further measurements and adjustments. Except for items 13 and 14, the number of factors extracted and the attributions of the items were very similar to those of the original scale, indicating that the PCSCCS-M was consistent with the original theoretical structure.

In addition, the PCSCCS-M demonstrated acceptable concurrent validity, which reflects the relationship between two scales for an identical feature. A statistically significant correlation between the PCSCCS-M and the C-SCGS (r = 0.339, *p* < 0.01) was found, although the correlation was weak according to general criteria [28]. Therefore, we conclude that this scale was sufficiently sensitive to evaluate the same feature as the C-SCGS.

Regarding the translation of the PCSCCS-M, most items appeared to have culturally equivalent terms in simplified Chinese, and we were able to switch between simplified and traditional characters without much further adaptation. For example, nurses from both Taiwan and mainland China believe that companionship and communication knowledge is important and is met with considerable enthusiasm when caring for patients; thus, item 1 (I know the basic knowledge of companionship and communication very well) and item 8 (I am passionate about caring for patients) had high scores (mean > 4.0). For cultural adaptation, we used the Chinese word “心灵关怀” in place of “灵性照护” in some sentences according to professionals and nurses. However, some differences in cultural components remain between Taiwan and mainland China. The most obvious difference is related to religion. Most nurses in Taiwan have religious beliefs. The PCSCCS was also developed based on caregivers with religious beliefs [22]. However, most nurses in mainland China do not believe in any religion [36], as reflected by the difference in the scale entries, with a lower score for item 18 (I am able to help patients build relationships with the Most High) for the mainland nurses and a higher score in Taiwan. According to the subsequent interviews, in the mainland, some nurses did not understand the meaning of “the Supreme”, and the researchers needed to explain this concept before the nurses could complete the questionnaire. Although the study suggests that one’s religion has a minor effect on how he or she understands spirituality and religion, spirituality is an important part of most religions [37–41]. This point warrants greater attention in further research. Additionally, in the mainland version of the scale, the measurement results showed that the scores for the items of Factor 1 (knowledge and skills of spiritual care) were higher than those for the Factor 3 items (spiritual care that meets spiritual needs), indicating that mainland nurses are more familiar with spiritual care knowledge but their practical ability and skills are insufficient. The opposite measurement results were found in Taiwan, possibly due to differences in nursing education between mainland China and Taiwan regarding spiritual care. Currently, nursing education in mainland China still lacks relevant spiritual care training, thus compromising nurses’ ability to provide spiritual care to patients [42]. The earliest spiritual care education program in China, which was proposed by Wu Zhenmin in 1982, began in Taiwan, and a spiritual care team that provides spiritual care has been formed in Taiwan [43]. However, studies on spiritual care education in mainland China have not been reported.

## Study limitations and directions for future research

The main shortcoming of the present study may be that the sample of nurses was mainly from the Henan and Jilin provinces of China. Therefore, the findings may not represent the opinions of all nurses in China.

The study has some implications for future research. The C-SCGS was selected to evaluate the concurrent validity of the PCSCCS-M. The topic of the C-SCGS is consistent with that of the current PSCSSC-M. The three domains of the PCSCCS-M were concurrently valid compared to most of the C-SCGS domains. However, the PCSCCS-M knowledge and skills domain did not correlate well with the C-SCGS spirituality perspectives domain (see Additional file 1: Table S5), indicating the need to further explore their relationships. Future studies may attempt to use structural equation modeling (SEM) to further analyze the factors influencing nurses’ spiritual care perceptions, their competencies and their relationships. Second, the study findings provide further support for the reliability and validity of the PCSCCS-M and its use as a measurement tool for nurses’ competencies in spiritual care. In addition, the tests of the PCSCCS-M across diverse study populations using CFA reveal a well-fitting model. However, the modification indices and the better model fit of the indicators of the adjusted model compared to the original model (see Additional file 1: Table S6) revealed that this three-subscale model may have multiple collinearity due to cross-loadings, which requires further modification (see Additional file 2: Figure S3). Future research should use exploratory structural equation modeling (ESEM) [44], which allows integration of features of CFA, SEM, and EFA in a single framework, to overcome some limitations of CFA.

## Relevance for practice

The PCSCCS-M can be used in nurses who work in different departments even though it was first designed for use with Christian palliative care professionals. This is consistent with the view that spiritual care extends beyond religion and is widely applicable [45]. For the education and training sector, such an assessment can offer important information regarding areas where nurses are not qualified and should receive education and training, which is thought to be essential to improving the spiritual care ability of nurses [46, 47]. For practical purposes, this evaluation will allow nurses to explore available resources to improve their abilities in providing care to meet patients’ spiritual needs. Although the PCSCCS was first designed based on data collected from trainees who received palliative care spiritual care training conducted by the SCMA, it showed sound psychometric properties in assessing the spiritual care capacities of nurses who are nonreligious. Future studies can test whether this instrument is useful with other health care workers (physicians and social workers, for example).

## Conclusions

The results of the present study showed that the PCSCCS-M is a potentially helpful instrument to measure mainland Chinese nurses’ competence in the delivery of spiritual care. Its application warrants further investigation.

## Additional files

Additional file 1: **Table S1**. Social and demographic information of the participants (*n* = 356). **Table S2**. Item analysis (n = 356). **Table S3**. Internal consistency of the PCSCCS-M (n = 356). **Table S4**. Rotated factor analysis of the PCSCCS-M (n = 356). **Table S5**. Cronbach’s alpha and Pearson’s Product-Moment Correlation between the Two Scales (PCSCCS-M & C-SCGS). **Table S6**. Model fit indices of the confirmatory factor analysis of the three- and six-factor models. (DOC 169 kb)

Additional file 2: **Figure S1**. Scree plot. **Figure S2**. Modified CFA of the three-factor 17-item model. **Figure S3**. Modified CFA of the three-factor 17-item model. **Figure S4**. Modified CFA of the three-factor 16-item model. (DOC 379 kb)

## Abbreviations

CFA: Confirmatory factor analysis
CR: The criterial value
C-SCCS: The Chinese version of the Spiritual Care Competency Scale
C-SCGS: The Chinese version of the Spiritual Care-Giving Scale
CVI: The content validity of an item
EFA: Exploratory factor analysis
ESEM: Exploratory structural equation modeling
KMO: Kaiser–Meyer–Olkin
PCSCCS: The Palliative Care Spiritual Care Competency Scale
PCSCCS-M: The Mainland Chinese-version Palliative Care Spiritual Care Competency Scale
SCGS: The Spiritual Care-Giving Scale
SEM: Structural Equation Model
SSCRS: The Spirituality and Spiritual Care Rating Scale
TVI: The Translation Validity Index
