# Supplementary material for: Psychometric properties of the Chinese mainland version of the Palliative Care Spiritual Care Competency Scale (PCSCCS-M) in nursing: a cross-sectional study
Source: BMC Palliat Care. 2019 Mar 8;18:27. doi: 10.1186/s12904-019-0409-6 (PMC6408799; doi:10.1186/s12904-019-0409-6)
Supplement: Supplementary file 1 — Table S1. Social and demographic information of the participants (n = 356). Table S2. Item analysis (n = 356). Table S3. Internal consistency of the PCSCCS-M (n = 356). Table S4. Rotated factor analysis of the PCSCCS-M (n = 356). Table S5. Cronbach’s alpha and Pearson’s Product-Moment Correlation between the Two Scales (PCSCCS-M & C-SCGS). Table S6. Model fit indices of the confirmatory factor analysis of the three- and six-factor models. (DOC 169 kb) [file 12904_2019_409_MOESM1_ESM.doc]

**Table S1** Social and demographic information of the participants (n＝356)

| **Variable** | **Number** | **Percent** |
| --- | --- | --- |
| **Gender** |  |  |
| Male | 20 | 5.6 |
| Female | 336 | 94.4 |
| **Age, years** |  |  |
| ≥18 | 64 | 18.0 |
| ≥26 | 116 | 32.6 |
| ≥31 | 132 | 37.1 |
| ≥41 | 39 | 11.0 |
| ≥51 | 5 | 1.4 |
| **Marital status** |  |  |
| Unmarried | 105 | 29.5 |
| Married | 245 | 68.8 |
| Divorced | 5 | 1.4 |
| Widowed | 1 | .3 |
| **Education** |  |  |
| Secondary vocational schools | 2 | .6 |
| Junior college | 68 | 19.1 |
| Undergraduate | 261 | 73.3 |
| Postgraduate or above | 25 | 7.0 |
| **department** |  |  |
| Internal medicine | 123 | 34.6 |
| Surgical | 63 | 17.7 |
| Pediatric | 14 | 3.9 |
| Obstetrics and gynecology | 29 | 8.1 |
| Emergency | 13 | 3.7 |
| ICU | 12 | 3.4 |
| The operating room | 4 | 1.1 |
| Outpatient service | 14 | 3.9 |
| Psychiatric | 41 | 11.5 |
| Others | 43 | 12.1 |
| **Working years, years (M ± SD)** | 10.4 | (8.8) |

**Table S2** Item analysis (n＝356)

| **PCSCCS-M items** | **Mean** | **SD** |  | **Extreme group comparison** |  | **Item-total correlations** | |  | **Homogeneity test** | | |  | **Numbers of substandard indicators** | **Notes** |
| --- | --- | --- | --- | --- | --- | --- | --- | --- | --- | --- | --- | --- | --- | --- |
|  |  |  |  | **Criterial ratio (CR)** |  | **Item-total correlation** | **Adjusted item-total correlation** |  | **Cronbach's α if item deleted** | **Communalities** | **Factor loading** |  |  |  |
| Q1 | 4.03 | 0.627 |  | 9.351** |  | 0.605** | 0.554 |  | 0.920 | 0.389 | 0.624 |  | 0 | retained |
| Q2 | 3.87 | 0.729 |  | 10.348** |  | 0.622** | 0.563 |  | 0.920 | 0.381 | 0.617 |  | 0 | retained |
| Q3 | 3.72 | 0.842 |  | 15.559** |  | 0.727** | 0.673 |  | 0.917 | 0.518 | 0.719 |  | 0 | retained |
| Q4 | 3.89 | 0.681 |  | 12.327** |  | 0.709** | 0.664 |  | 0.918 | 0.499 | 0.707 |  | 0 | retained |
| Q5 | 4.04 | 0.589 |  | 8.865** |  | 0.649** | 0.605 |  | 0.919 | 0.441 | 0.664 |  | 0 | retained |
| Q6 | 3.91 | 0.668 |  | 13.543** |  | 0.760** | 0.722 |  | 0.916 | 0.608 | 0.780 |  | 0 | retained |
| Q7 | 4.08 | 0.581 |  | 7.360** |  | 0.627** | 0.581 |  | 0.920 | 0.435 | 0.660 |  | 0 | retained |
| Q8 | 4.11 | 0.573 |  | 10.834** |  | 0.710** | 0.673 |  | 0.918 | 0.555 | 0.745 |  | 0 | retained |
| Q9 | 4.04 | 0.642 |  | 10.755** |  | 0.727** | 0.687 |  | 0.917 | 0.582 | 0.763 |  | 0 | retained |
| Q10 | 4.03 | 0.627 |  | 11.238** |  | 0.730** | 0.691 |  | 0.917 | 0.569 | 0.754 |  | 0 | retained |
| Q11 | 3.84 | 0.701 |  | 16.791** |  | 0.802** | 0.768 |  | 0.915 | 0.675 | 0.821 |  | 0 | retained |
| Q12 | 3.94 | 0.645 |  | 13.378** |  | 0.752** | 0.715 |  | 0.917 | 0.599 | 0.774 |  | 0 | retained |
| Q13 | 3.57 | 0.848 |  | 8.032** |  | 0.494** | 0.411 |  | 0.925 | **0.163** | **0.404** |  | **2** | **retained** |
| Q14 | 3.28 | 0.989 |  | 6.720** |  | **0.389**** | **0.280** |  | **0.931** | **0.076** | **0.275** |  | **5** | **deleted** |
| Q15 | 3.83 | 0.660 |  | 12.334** |  | 0.710** | 0.666 |  | 0.918 | 0.497 | 0.705 |  | 0 | retained |
| Q16 | 3.87 | 0.610 |  | 12.135** |  | 0.728** | 0.690 |  | 0.917 | 0.537 | 0.733 |  | 0 | retained |
| Q17 | 3.83 | 0.620 |  | 12.950** |  | 0.704** | 0.662 |  | 0.918 | 0.496 | 0.704 |  | 0 | retained |
| Q18 | 3.71 | 0.716 |  | 15.452** |  | 0.718** | 0.671 |  | 0.917 | 0.492 | 0.701 |  | 0 | retained |
| standard |  |  |  | ≧3.000 |  | 0.400 | 0.400 |  | 0.923^a^ | 0.200 | 0.450 |  |  | retained |

**. p＜0,01. a: The Cronbach's α of the PCSCCS-M was 0.923; Guttman Split-Half coefficient＝0.855; Spearman-Brown coefficient＝0.857;

|  |
| --- |

**Table S3** Internal consistency of PCSCCS-M (n＝356)

| **PCSCCS-M items** | **Mean** | **SD** | **Item-total correlation** | **Adjusted item-**  **total correlation** | **Cronbach's α if item deleted** | **Communalities** | **Factor loading** |
| --- | --- | --- | --- | --- | --- | --- | --- |
| Q1 | 4.03 | 0.627 | 0.631** | 0.580 | 0.929 | 0.397 | 0.630 |
| Q2 | 3.87 | 0.729 | 0.633** | 0.573 | 0.929 | 0.383 | 0.619 |
| Q3 | 3.72 | 0.842 | 0.735** | 0.680 | 0.927 | 0.518 | 0.720 |
| Q4 | 3.89 | 0.681 | 0.712** | 0.666 | 0.927 | 0.498 | 0.706 |
| Q5 | 4.04 | 0.589 | 0.659** | 0.614 | 0.928 | 0.443 | 0.666 |
| Q6 | 3.91 | 0.668 | 0.773** | 0.735 | 0.925 | 0.611 | 0.782 |
| Q7 | 4.08 | 0.581 | 0.651** | 0.606 | 0.928 | 0.443 | 0.666 |
| Q8 | 4.11 | 0.573 | 0.730** | 0.694 | 0.927 | 0.562 | 0.750 |
| Q9 | 4.04 | 0.642 | 0.751** | 0.712 | 0.926 | 0.591 | 0.768 |
| Q10 | 4.03 | 0.627 | 0.747** | 0.708 | 0.926 | 0.574 | 0.757 |
| Q11 | 3.84 | 0.701 | 0.813** | 0.780 | 0.924 | 0.676 | 0.822 |
| Q12 | 3.94 | 0.645 | 0.767** | 0.730 | 0.925 | 0.603 | 0.776 |
| Q13 | 3.57 | 0.848 | 0.437** | 0.344 | 0.937 | 0.148 | 0.385 |
| Q15 | 3.83 | 0.660 | 0.704** | 0.658 | 0.927 | 0.491 | 0.701 |
| Q16 | 3.87 | 0.610 | 0.728** | 0.689 | 0.926 | 0.534 | 0.731 |
| Q17 | 3.83 | 0.620 | 0.701** | 0.658 | 0.927 | 0.492 | 0.701 |
| Q18 | 3.71 | 0.716 | 0.702** | 0.652 | 0.927 | 0.482 | 0.694 |

Cronbach's α ＝ 0.931; Guttman Split-Half coefficient＝0.862; Spearman-Brown coefficient＝0.865.

**Table S4** Rotated factor analysis of the PCSCCS-M (n＝356)

| **PCSCCS-M (Chinese Mainland version)^a^** |  | **Factor** | | |  | **C^2^** |
| --- | --- | --- | --- | --- | --- | --- |
|  |  | **1** | **2** | **3** |  |  |
| **Factor 1 灵性关怀知能 (**knowledge and skills of spiritual care**)** |  |  |  |  |  |  |
| Q1我清楚陪伴与沟通的基本知识 (I know the basic knowledge of companionship and communication very well) |  | **0.551** | 0.417 | 0.150 |  | 0.500 |
| Q2我具备临终关怀与护理的知识 (I have knowledge of hospice care and care) |  | **0.794** | 0.120 | 0.253 |  | 0.709 |
| Q3我熟悉医疗、社会、心理、心灵照顾的专业资源 (I am familiar with professional resources for medical, social, psychological and spiritual care) |  | **0.760** | 0.290 | 0.267 |  | 0.732 |
| Q4我对自己的特质有觉察与认识 (I have awareness and understanding of my own traits) |  | **0.641** | 0.396 | 0.227 |  | 0.619 |
| **Factor 2 灵性关怀自我觉察 (**self-awareness and attitude towards spiritual care**)** |  |  |  |  |  |  |
| Q5我能反省自己助人的动机 (I can reflect on my motivation to help others) |  | 0.423 | **0.637** | 0.076 |  | 0.591 |
| Q6我对从事心灵关怀有使命感 (I have a sense of mission to engage in spiritual care) |  | 0.411 | **0.603** | 0.324 |  | 0.638 |
| Q7我认为心灵关怀工作要符合专业与社会规范 (I believe that spiritual care work must conform to professional and social norms) |  | 0.222 | **0.719** | 0.158 |  | 0.590 |
| Q8我对关怀患者有热忱 (I am passionate about caring for patients) |  | 0.210 | **0.779** | 0.246 |  | 0.712 |
| Q9我会主动关心患者/家属的心灵需求 (I will take the initiative to care about the spiritual needs of patients/families) |  | 0.164 | **0.753** | 0.346 |  | 0.714 |
| Q10我对自己的人生经历有相当的了解、反省与思考 (I have a good understanding and reflection on my life experience) |  | 0.180 | **0.742** | 0.326 |  | 0.689 |
| **Factor 2 灵性关怀照顾 (**spiritual care that meets patient’s spiritual needs**)** |  |  |  |  |  |  |
| Q11我能够陪伴或协助家属走过悲伤失落 (I can accompany or assist my family to walk through sadness and loss) |  | 0.231 | 0.556 | **0.601** |  | 0.724 |
| Q12我能够协助患者接纳他人、与他人和好 (I can help patients to accept others and be nice with others) |  | 0.232 | 0.546 | **0.532** |  | 0.635 |
| Q13协助患者体会宽恕与被宽恕的经验对我是困难的 (It was difficult for me to assist the patient with the experience of forgiveness and being forgiven) |  | -0.054 | 0.192 | **0.496** |  | 0.286 |
| Q15我能够协助患者获得爱与被爱的经验（体验）(I can help patients gain the experience of love and being loved) |  | 0.275 | 0.212 | **0.737** |  | 0.663 |
| Q16我能够陪伴或协助患者/家属共同面对核心的议题 (I can accompany or assist patients/families to face core issues together) |  | 0.316 | 0.239 | **0.722** |  | 0.679 |
| Q17我能够洞察患者/家属的心灵需求 (I am able to gain insight into the spiritual needs of patients/families) |  | 0.316 | 0.205 | **0.710** |  | 0.647 |
| Q18我能够协助患者建立与至高者（他人、更大的力量或自然界）的关系 (I am able to help patients build relationships with the Most High (others, greater power or nature)) |  | 0.283 | 0.150 | **0.789** |  | 0.724 |
| Eigen value |  | 8.446 | 1.346 | 1.060 |  |  |
| Percentage of variance (total: 63.839) |  | 49.684 | 7.917 | 6.238 |  |  |

**Notes:** Extraction method: principal-component analysis with varimax rotation. Item with a factor loading greater than 0.40 is retained for that factor. C^2^ indicates communality coefficients. ^a^: Cronbach's α ＝ 0.931; KMO ＝ 0.936, Bartlett's Test of Sphericity：Approximate χ2＝3625.695, df＝136, p＝0.000.

**Table S5 Cronbach’s alpha and Pearson’s Product-Moment Correlation between the Two Scales (PCSCCS-M & C-SCGS).**

| **Measures** | **PCSCCS-M** | **Factor 1** | **Factor 2** | **Factor 3** | **C-SCGS** | **C-SCGS 1** | **C-SCGS 2** | **C-SCGS 3** | **C-SCGS 4** |
| --- | --- | --- | --- | --- | --- | --- | --- | --- | --- |
| **PCSCCS-M** | α＝0.931 |  |  |  |  |  |  |  |  |
| **Factor 1** | 0.848^**^ | α＝0.811 |  |  |  |  |  |  |  |
| **Factor 2** | 0.898^**^ | 0.690** | α＝0.889 |  |  |  |  |  |  |
| **Factor 3** | 0.910^**^ | 0.650** | 0.703** | α＝0.868 |  |  |  |  |  |
| **C-SCGS** | 0.340** | 0.236** | 0.382** | 0.280** | α＝0.966 |  |  |  |  |
| **C-SCGS 1** | 0.366** | 0.274** | 0.404** | 0.294** | 0.930** | α＝0.941 |  |  |  |
| **C-SCGS 2** | 0.359** | 0.250** | 0.350** | 0.338** | 0.874** | 0.756** | α＝0.852 |  |  |
| **C-SCGS 3** | 0.118* | 0.071^a^ | 0.182** | 0.065^a^ | 0.772** | 0.627** | 0.531** | α＝0.836 |  |
| **C-SCGS 4** | 0.256** | 0.152** | 0.325** | 0.197** | 0.899** | 0.752** | 0.705** | 0.734** | α＝0.866 |

Pearson’s correlation coefficient test was used, two-tailed. Cronbach’s alpha on the diagonal in parenthesis. Simplified Chinese version of Palliative Care Spiritual Care Competency Scale (PCSCCS-M): knowledge and skills of spiritual care (Factor 1), self-awareness and attitude of spiritual care (Factor 2), and spiritual care that meets the spiritual needs (Factor 3). Chinese version of the Spiritual Care-Giving Rating Scale (C-SCGS): Attributes for Spiritual Care (C-SCGS 1), definitions of spirituality and spiritual care (C-SCGS 2), spiritual perspectives (C-SCGS 3), spirituality and spiritual care values(C-SCGS 4).

a :p > 0.05; * p < 0.01; ** p < 0.01

**Table S6** Model fit indices of the confirmatory factor analysis of the three- and six-factor models

| **Global model fit index** | **Acceptable criterion** | **3-factor model** | **Adjusted 3-factor model ^a^** | **Adjusted 3-factor model ^b^** |
| --- | --- | --- | --- | --- |
| Absolute fit index |  |  |  |  |
| Likelihood-ratio x^2^ |  | 428.257 | 221.681 | 203.740* |
| Degree of freedom |  | 116 | 104 | 92 |
| Goodness of fit index (GFI) | ≥ 0.90 | 0.876 | 0.936 | 0.937 |
| Adjusted goodness of fit index (AGFI) | ≥ 0.90 | 0.837 | 0.906 | 0.907 |
| Root mean square residual (RMR) | ≤ 0.08 | 0.026 | 0.019 | 0.015 |
| Standardized root mean squre error of approximation (RMSEA) | ≤ 0.08 | 0.087 | 0.057 | 0.059 |
| Relative fit index |  |  |  |  |
| Normed fit index (NFI) | ≥ 0.90 | 0.873 | 0.934 | 0.938 |
| No normed fit index (NNFI, TLI) | ≥ 0.90 | 0.887 | 0.952 | 0.954 |
| Relative fit index (RFI) | ≥ 0.90 | 0.851 | 0.914 | 0.919 |
| Incremental fit index (IFI) | ≥ 0.90 | 0.904 | 0.964 | 0.965 |
| Comparative fit index (CFI) | ≥ 0.90 | 0.903 | 0.964 | 0.965 |
| Parsimony fit index |  |  |  |  |
| Parsimony goodness of fit index (PGFI) | ≥ 0.50 | 0.853 | 0.765 | 0.767 |
| Parsimony normed fit index (PNFI) | ≥ 0.50 | 0.744 | 0.714 | 0.719 |
| Parsimony comparative fit index (PCFI) | ≥ 0.50 | 0.770 | 0.737 | 0.740 |
| Likelihood-ratio χ2/degree of freedom | ≤ 3 | 3.692 | 2.132 | 2.215 |

*p < 0.001

^a^: adjusted model with 17 items; ^b^: with 16 items by deleting the item 13
