# Supplementary material for: Psychometric properties of the Chinese mainland version of the Palliative Care Spiritual Care Competency Scale (PCSCCS-M) in nursing: a cross-sectional study
Source: BMC Palliat Care. 2019 Mar 8;18:27. doi: 10.1186/s12904-019-0409-6 (PMC6408799; doi:10.1186/s12904-019-0409-6)
Supplement: Supplementary file 2 — Figure S1. Scree plot. Figure S2. Modified CFA of the three-factor 17-item model. Figure S3. Modified CFA of the three-factor 17-item model. Figure S4. Modified CFA of the three-factor 16-item model. (DOC 379 kb) [file 12904_2019_409_MOESM2_ESM.doc]

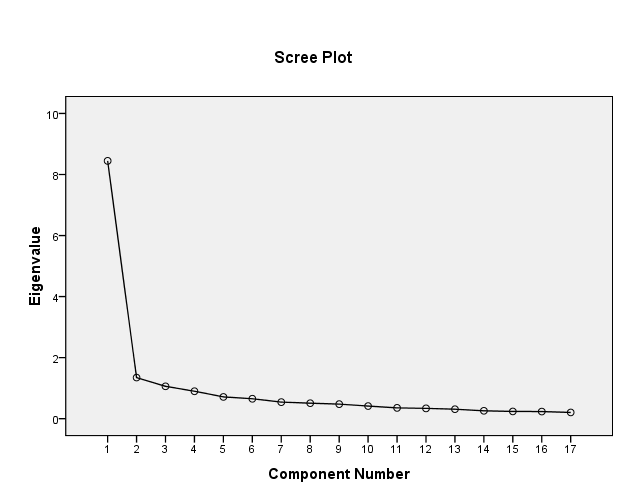


Number of components

**Figure S1.** Scree plot


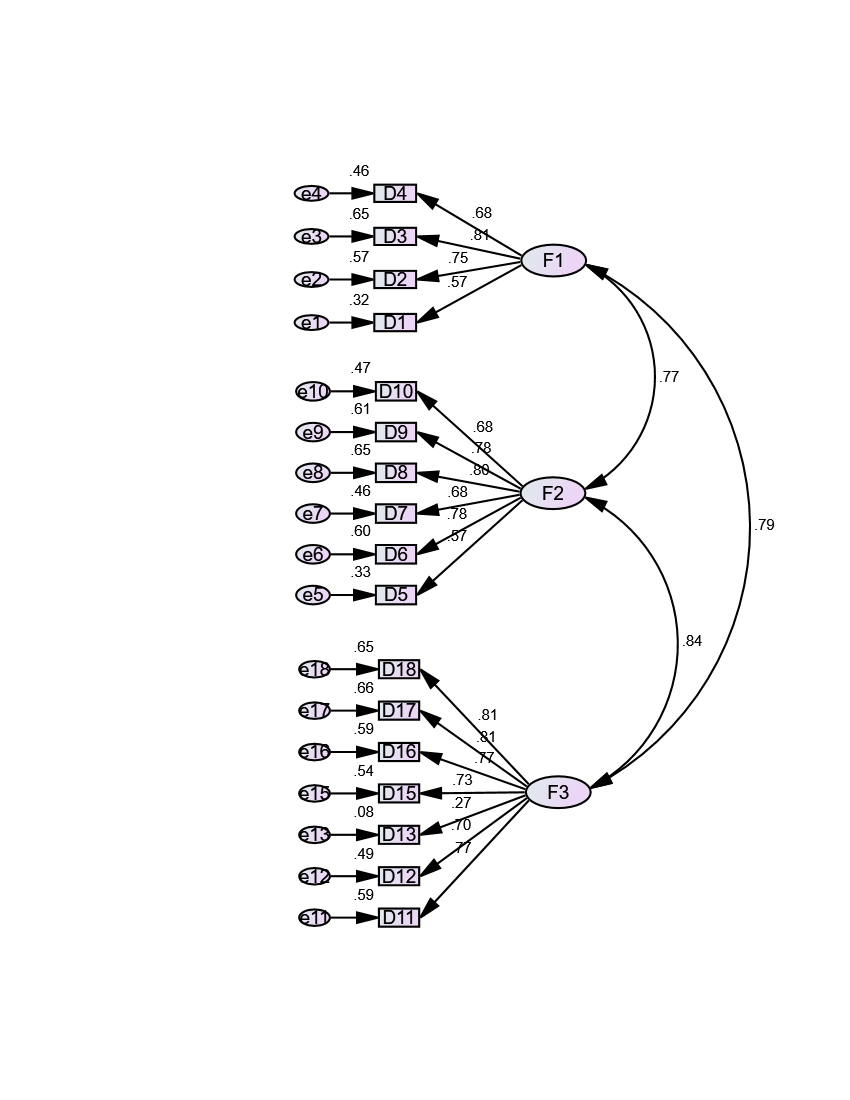


**Figure S2.** Modified CFA of the three factor 17-item model


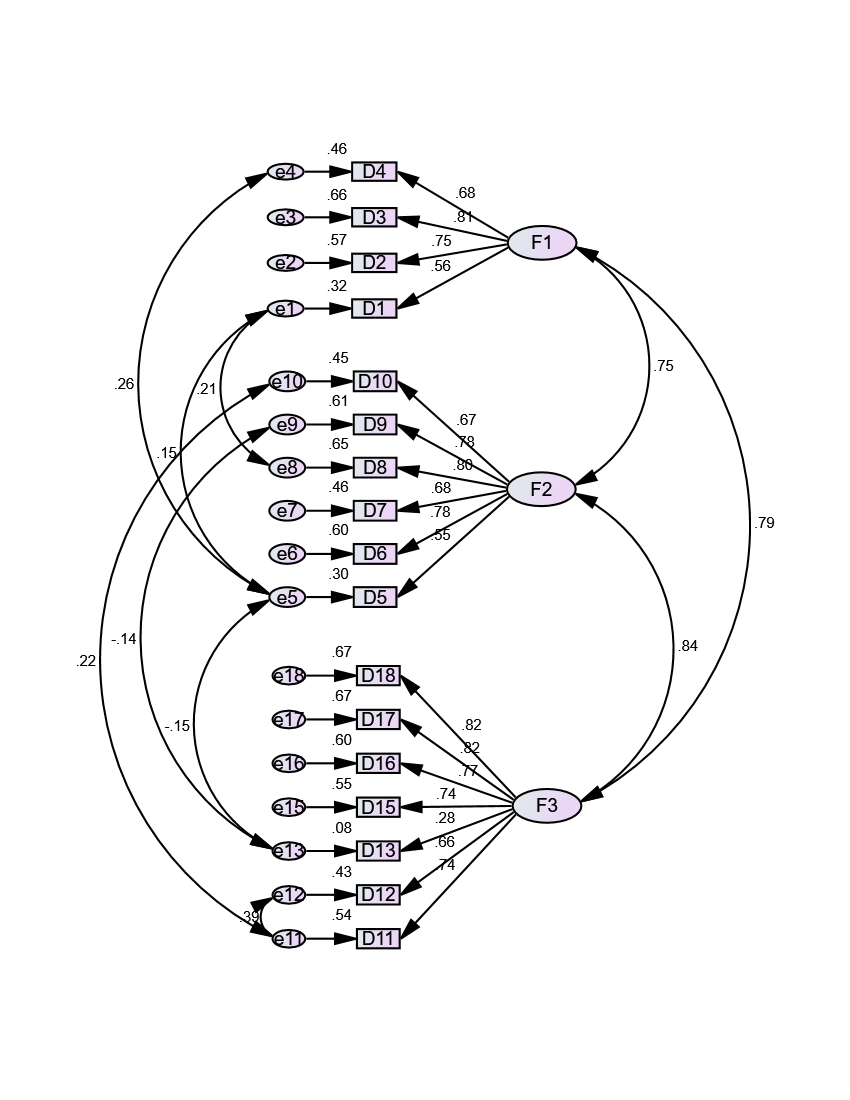


**Figure S3.** Modified CFA of the three factor 17-item model


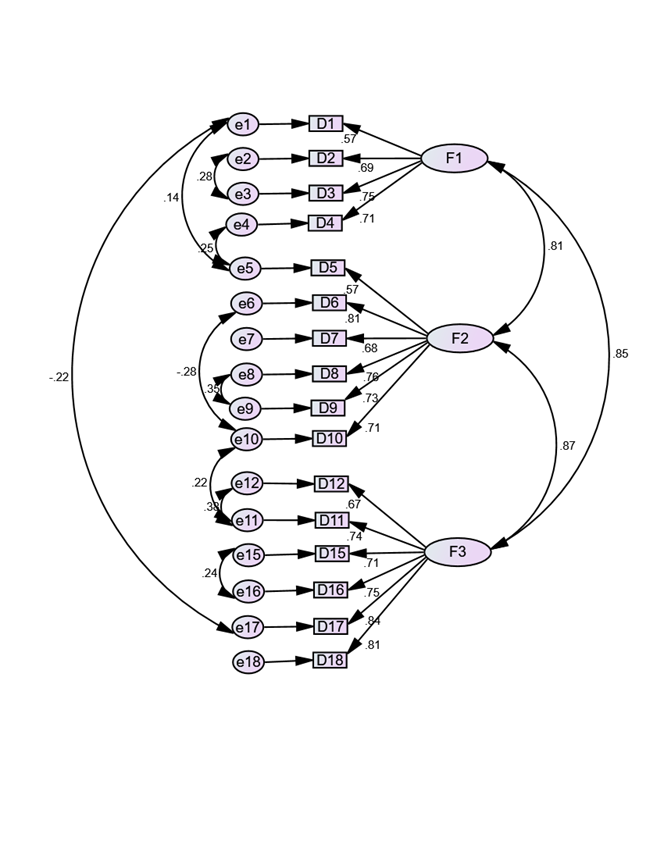


**Figure S4.** Modified CFA of the three factor 16-item model
